# *Monomorium dryhimi* sp. n., a new ant species (Hymenoptera, Formicidae) of the *M. monomorium* group from Saudi Arabia, with a key to the Arabian *Monomorium monomorium*-group

**DOI:** 10.3897/zookeys.106.1390

**Published:** 2011-06-15

**Authors:** Abdulrahman S. Aldawood, Mostafa R. Sharaf

**Affiliations:** Plant Protection Department, College of Food and Agriculture Sciences, King Saud University, Riyadh 11451, P. O. Box 2460, Saudi Arabia

**Keywords:** Myrmicinae, Arabia, new species, alpha taxonomy, Palaearctic region, Asir Province, endemism

## Abstract

A new ant species, *Monomorium dryhimi*, is described based on workers from a single colony collected in Al Bahah, Asir Province, Saudi Arabia. This is the fourth species of the *Monomorium monomorium*-group collected from Arabian Peninsula, and appears to be closely related to *Monomorium holothir* Bolton, 1987, from Kenya. It can be distinguished by the following characters: head in profile with a weakly convex dorsal surface and a clearly convex ventral surface; eyes of moderate size with maximum diameter EL 0.19–0.25 × HW and with 6 ommatidia in the longest row; body colour yellow to light brownish yellow. In some individuals, head and gaster slightly but conspicuously darker than rest of body. Second halves of first and second gastral tergites with two characteristic brownish transverse bands. An identification key to the workers of the Arabian species of the *Monomorium monomorium*-group is presented. Scanning electron micrographs are given to illustrate the new species.

## Introduction

The ant genus *Monomorium* was established by Mayr (1855) for the newly described species *Monomorium minutum* Mayr (which was given the new name *Monomorium monomorium* Bolton (1987:287)). This genus includes more than 300 species and subspecies (Bolton 1995; Bolton et al. 2007) found in all zoogeographic regions with most species occurring in the Old World tropics and temperate regions (Brown 2000). Taxonomic revisions of the Australian and Malagasy *Monomorium* fauna were carried out by (Heterick (2001, 2006) respectively. The Afrotropical *Monomorium* fauna was comprehensively revised by Bolton (1987). Two new South American species of *Monomorium* were described by Fernández (2007) and notes on the genus were presented. The *Monomorium* fauna of Arabian Peninsula was reviewed and listed giving 53 species for the region (Collingwoood and Agosti 1996). Most species of *Monomorium* nest in rotten wood, under stones, or directly in the soil.

Members of the genus *Monomorium* can be distinguished by the following characters: monomorphic to polymorphic; antennae 10–12 segmented (most frequently 12), usually with a conspicuous 3-segmented club; mandibles with 3–5 teeth, median clypeal seta conspicuous; median portion of clypeus raised, the raised section longitudinally bicarinate; the carinae usually distinct; metanotal groove present, commonly impressed; propodeal dorsum usually unarmed and rounding into the declivity; propodeal spiracle usually circular, located at about the midlength of the sclerite (Bolton 1987).

The *Monomorium monomorium*-group (Bolton 1987) can be distinguished by the following characters: Monomorphic; mandibles unsculptured; the masticatory margin usually with 4 teeth; palp formula predominantly 2,2; cephalic dorsum unsculptured and glossy smooth except for scattered hair-pits; eyes always present, size small to large (0.15–0.38×HW), with 4 or more ommatidia in the longest row; head always longer than broad (CI 72–89); metanotal groove moderately to strongly impressed, with distinct cross-ribs; propodeal dorsum rounding into declivity, not angulate or dentate; petiole, postpetiole and gaster usually unsculptured.

The first treatment of the Arabian *Monomorium* fauna was Collingwood’s (1985) study of the genus in Saudi Arabia where 20 species were recorded, of which a single species was of the *monomorium*-group, *Monomorium clavicorne* Andre, 1881. The second and more comprehensive contribution was that of Collingwood and Agosti (1996) for the *Monomorium* in the Arabian Peninsula. In that study, 53 species were recorded, 17 of which were described from Saudi Arabia including two species belonging to the *Monomorium monomorium*-group, *Monomorium montanum* and *Monomorium qarahe*. Since that time the contributions to the Arabian *Monomorium* fauna were descriptions of only two new species, *Monomorium nimihil* Collingwood from Socotra Archipelago (Collingwood et al. 2004) and *Monomorium moathi* Sharaf & Collingwood from Yemen (Aldawood et al. 2010).

In the present paper a new species of the genus *Monomorium*, *Monomorium dryhimi* is described from Saudi Arabia based on worker caste. A key to the four Arabian species of the *Monomorium monomorium*-group is presented.

## Materials and methods

All measurements are in millimeters and follow the standard measurements (Bolton 1987).

TL	Total Length; the outstretched length of the ant from the mandibular apex to the gastral apex.

HW	Head Width; the maximum width of the head behind eyes in full-face view.

HL	Head Length; the maximum length of the head, excluding the mandibles.

CI	Cephalic Index (HW × 100/HL).

SL	Scape Length, excluding basal neck.

SI	Scape Index (SL × 100/HW).

EL	Eye Length; the maximum diameter of the eye.

ML	Mesosoma Length; the length of the mesosoma in lateral view, from the point at which the pronotum meets the cervical shield to the posterior base of the propodeal lobes or teeth.

PRW	Pronotal width, maximum width in dorsal view.

PL	Petiole Length; the maximum length measured in dorsal view, from the anterior margin to the posterior margin.

PW	Petiole Width; maximum width measured in dorsal view.

PPL	Postpetiole Length; maximum length measured in dorsal view.

PPW	Postpetiole Width; maximum width measured in dorsal view.

**Figures 1–7.**
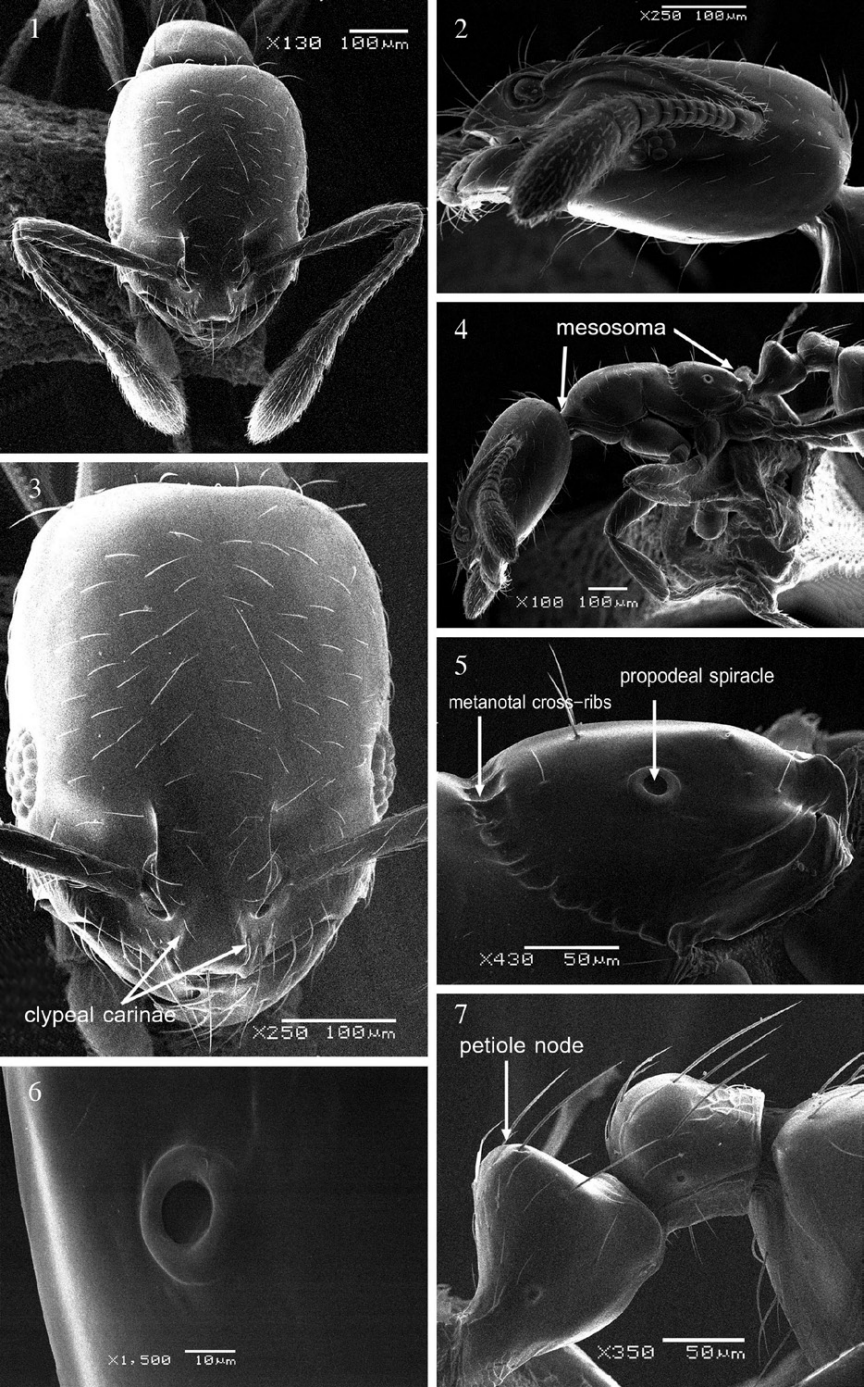
*Monomorium dryhimi* sp.n., paratype worker **1, 3** Head in full-face view **2** head in profile **4** body in profile **5** propodeum **6** propodeal spiracle **7** petiole and postpetiole.

Images taken under the scanning electron microscope ((SEM) JSM-6380 LA) were used to record morphological details of the new species (Figs 1–7).

## Results

### Monomorium dryhimi

Aldawood & Sharaf sp. n.

urn:lsid:zoobank.org:act:4C171A6D-B1F5-4D4D-BBAD-CE2250167E5B

http://species-id.net/wiki/Monomorium_dryhimi

Figs 1–7 

#### Holotype worker.

TL1.84, HL 0.48, HW 0.34, SL 0.31, ML 0.46, EL 0.08, PRW 0.22, PL 0.14, PW 0.11, PPL 0.08, PPW 0.11, SI 91, CI 71.

#### Paratypes.

TL 1.42–1.84, HL 0.42–0.49, HW 0.32–0.36, SL 0.26–0.32, ML 0.39–0.46, EL 0.07–0.08, PRW 0.19–0.24, PL 0.09–0.14, PW 0.08–0.11, PPL 0.05–0.09, PPW 0.09–0.12, SI 74–91, CI 73–83.(N=13).

#### Holotype worker.

SAUDI ARABIA, Al Bahah province, Amadan forest, Al Mandaq governorate, 20°12'N, 41°13'E, 1881 m.a.s.l. 19.V.2010 *(M. R. Sharaf & A. S. Aldawood Leg.)*; King Saud Museum of Arthropods (KSMA), College of Food and Agriculture Sciences, King Saud University, Riyadh, Kingdom of Saudi Arabia.

#### Paratypes.

27 workers, same locality and data as holotype; 1 deposited in the Muséum ďHistoire Naturelle, Geneva, Switzerland (Dr Bernhard Merz); 1 in Naturhistorisches Museum, Basel, Switzerland (Mrs. Isabelle Zürcher-Pfander); 1 in California Academy of Science (Dr Brian Fisher); 1 in World Museum Liverpool, Liverpool, U.K (Dr Guy Knight), 1 in The Natural History Museum, London (Mr. Barry Bolton); 15 workers, SAUDI ARABIA, Elqamh park, Belgershi, Al Bahah, 17.V.2010 *(M. R. Sharaf & A. S. Aldawood Leg.)* These paratypes are in the King Saud Museum of Arthropods, King Saud University, Riyadh, Saudi Arabia.

#### Worker.

Headdistinctly much longer than broad with weakly convex sides and straight or feebly concave posterior margin (Fig. 1). Underside of headwith several long hairs but not forming a psammophore (Fig. 2). Head in profile with a weakly convex dorsal surface and a distinctly convex ventral surface (Fig. 2). Clypeal carinae sharply developed and distinctly elevated, divergent anteriorly and reaching the anterior margin at a pair of short low triangular projecting angles (Fig. 3). The median portion of anterior clypeal margin clearly concave. Eyes oval and of moderate size (EL 0.19–0.25 × HW) with 6 ommatidia in the longest row (Figs 2, 3). With head in profile, eyes consist of a peripheral ring of ommatidia encircling two rows of ommatidia within the ring (Figs 2, 3). In lateral view, the maximum diameter of the eyes clearly greater than the distant between the anteriormost point of the eyes and the nearest point of the mandibular articulation. Frontal lobes farther apart. Antennal scapes, when laid straight back, fail to reach posterior margin (Fig. 1). Mesosoma in profilewith a flat promesonotal dorsum, which slopes posteriorly to a well developed metanotal groove (Fig. 4). Metanotal cross-ribs relatively long and distinct (Fig. 5). Propodeal spiraclessmall and pinhole-like (Fig. 6). Propodeal dorsum evenly sloping, the posterior third more strongly sloping than the anterior two-thirds (Fig. 5). Petiole nodehigh and narrowly subconical, narrowly rounded above (Fig. 7). Petiole peduncle short and stout with a distinct ventral process. Postpetiole node smaller, lower, and more broadly rounded than petiole. Petiole and postpetiole each with three pairs of long backward directed hairs. Body pilosity abundant, shorter on head dorsum. Anterior pronotal margin with two pairs of hairs, middle part of pronotum with a single pair, promesonotum with 3–4 pairs of hairs. Dorsum and declivity of propodeum each with one pair of hairs. Overall yellow to light brownish yellow. In many individuals head and gaster are slightly but conspicuously, darker than rest of body. Second halves of first and second gastral tergites with characteristic brownish transverse bands. Body smooth and shining.

#### Etymology.

This species is named in honor of Prof. Yousif N. Aldryhim, economic entomologist, Department of Plant Protection, College of Food and Agriculture Sciences, King Saud University, Kingdom of Saudi Arabia.

## Discussion

This new species is a member of the *Monomorium monomorium* group with closest resemblance to *Monomorium holothir* Bolton, 1987, which was described from Kenya. Both species sharing the following characters: clypeal carinae sharply developed and distinctly elevated; head sides behind eyes weakly convex; posterior margin feebly concave; in lateral view the maximum diameter of eyes clearly greater than the distance between anteriormost point of the eye and the nearest point of the mandibular articulation; body colour yellow to light brownish yellow; relatively similar body dimensions e.g. HL, HW, SI, and CI.

*Monomoroium dryhimi* can be easily separated from *Monomorium holothir* by the following characters: eyes relatively small, their maximum diameter EL 0.19–0.25 × HW and with 6 ommatidia in the longest row, while in *holothir* eyes larger, their maximum diameter EL 0.30 × HW and with 8–9 ommatidia in the longest row. In *Monomorium dryhimi*, the median portion of anterior clypeal margin is clearly concave, whereas it is transverse to feebly concave in *holothir*. Moreover, in *Monomorium dryhimi* head in profile with a weakly convex dorsal surface and a clearly convex ventral surface, whereas in *Monomorium holothir*, head in profile dorsoventrally flattened. Furthermore, the promesonotum in *Monomorium dryhimi* has 3–4 pairs of hairs whereas in *holothir* the promesonotum have 8 pairs of hairs.

### Biology of Monomorium dryhimi

**Figure 8.**
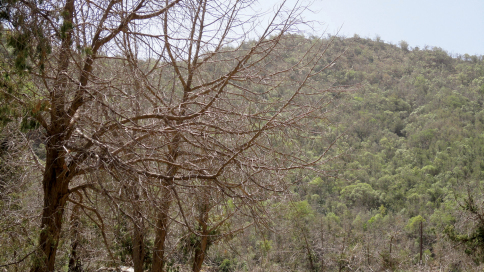
Type locality, Amadan forest, Al Mandaq governorate, Al Bahah province, Kingdom of Saudi Arabia.

The type locality is a forest called Amadan, Al Mandaq governorate, Al Bahah province, Kingdom of Saudi Arabia, with much wild vegetation including *Erica arborea* L, *Juniperus procera* Hochst. Ex Endle.and *Acacia gerrardii* Benth (Fig. 8). *Monomorium dryhimi* type was taken from a nest under a stone on hard-packed soil which contained tens of workers and was found in relatively elevated area of a valley which is high enough to avoid direct impacts of flooding. No additional nests were found despite extensive surveys. In addition, we were not able to collect foraging workers near the nest. It appears that members of the *Monomorium monomorium* group may prefer inhabiting areas of high elevations. All the four Arabian species were found inhabiting elevated localities with more than 1800 m asl., except *Monomorium clavicorne* which was also recorded from both relatively lower elevated areas including Riyadh and Al Qatif, in Central and Eastern regions of Saudi Arabia, respectively, and also from a much elevated area, Fayfa, Asir province (Collingwood 1985). Apparently this species is endemic to the chain of Asir Mountains which extends to Yemen.

## Key to the Arabian species of the Monomorium monomorium-group

**Table:** 

1	Antennae with 11 segments; terminal funicular segment broadly swollen	*Monomoroium clavicorne*
–	Antennae with 12 segments; terminal funicular segment enlarged, not swollen	2
2	Head, in full-face view, with long hairs surrounding posterior margin and head sides forming a fringe; metanotal groove shallow	*Monomoroium qarahe*
–	Head, in full-face view, without a fringe of long hairs; metanotal groove sharp and distinct	3
3	Larger yellow species; TL 1.70–2.30, HW 0.40; metanotal groove sharp but too small to break the dorsal outline; pronotum with a single pair of curved hairs	*Monomoroium montanum*
–	Smaller yellowish to light brownish yellow species, first and second gastral tergites with light brownish bands; TL 1.42–1.84; HW 0.32–0.36; metanotal groove sharp and distinctly breaks the dorsal outline; anterior pronotal margin with two pairs of hairs, middle part of pronotum with a single pair	*Monomoroium dryhimi* sp. n.

## Supplementary Material

XML Treatment for Monomorium dryhimi
